# The correlation between common 2D femoral notch parameters and 3D notch volume: a retrospective MRI study

**DOI:** 10.1186/s12891-019-2530-3

**Published:** 2019-04-06

**Authors:** Chengyuan Zhang, Xuancheng Zhang, Zhaoyi Fang, Feng Wang, Feng Yuan, Guoming Xie, Jinzhong Zhao

**Affiliations:** 1 Department of Orthopedics, Shanghai Sixth People’s Hospital East Affiliated to Shanghai University of Medicine and Health Sciences, Pudong New Area, Shanghai, 201306 People’s Republic of China; 2 Department of Sports Medicine, Shanghai Jiao Tong University Affiliated Sixth People’s Hospital, Xuhui District, Shanghai, 200233 China; 3 Department of Sports Medicine, Shanghai Sixth People’s Hospital East Affiliated to Shanghai University of Medicine and Health Sciences, Pudong New Area, Shanghai, 201306 People’s Republic of China

**Keywords:** ACL injury, Femoral notch, Volume, Width, Magnetic resonance imaging

## Abstract

**Background:**

Although the stenotic femoral intercondylar notch was associated with anterior cruciate ligament (ACL) injuries, the parameters for notch assessment were numerous. The present study aimed to compare the 2-dimensional (2D) femoral notch parameters, including the notch width (NW) and notch width index (NWI), with the 3-dimensional (3D) notch volume based on magnetic resonance imaging (MRI), to determine appropriate femoral parameters for ACL injuries.

**Methods:**

Two hundred forty individuals were included in this study, including 120 patients with ACL ruptures and 120 age- and gender-matched individuals without ACL ruptures. The NWs and NWIs were measured at four sites (the popliteal groove, the notch inlet and outlet, and the ACL attachment), and the notch volumes were calculated. The Pearson correlation coefficients between the 2D and 3D parameters were calculated. A multivariate analysis of the ACL injuries was conducted with these parameters and the demographic data.

**Results:**

The associations of the NW and NWI with the notch volume at each of the four locations of the femoral notch were poor in the subgroup analysis, with the exception of the NW in the male ACL-intact group (R = 0.307, 0.256, 0.404 and 0.387 at the popliteal groove, notch inlet and outlet, and ACL attachment, respectively). The multivariate analysis revealed that the notch volume (OR = 0.679, *P* < 0.001) and the NW at the popliteal groove (OR = 0.844, *P* = 0.004), notch inlet (OR = 0.720, P < 0.001) and ACL attachment (OR = 0.871, *P* = 0.028) were predictable parameters to the risk of ACL injuries.

**Conclusions:**

The correlations between the 2D parameters and the 3D volumes were weak. The notch volume and the NW at the popliteal groove, notch inlet and ACL attachment were useful parameters for predicting the risk of ACL injuries.

**Level of evidence:**

Level III, case-control study.

## Background

Decreased femoral intercondylar notch size is associated with an increased risk of anterior cruciate ligament (ACL) injuries, as reported by numerous studies [1]. This phenomenon is mainly due to the ACL impinging on the lateral femoral condyle within a stenotic notch when receiving anterior shear forces or tibial rotation [2–4]. Some scholars have suggested that small femoral notches are associated with thin and weak ACLs, which means that ACL injuries can easily occur [5–7].

Currently, a large number of parameters are used for femoral notch size assessment, most of which are 2-dimensional (2D) notch parameters [1, 7–11] The notch width (NW), the width between the inner wall of the lateral femoral condyle and the outer wall of the medial femoral condyle as measured at the level of the popliteal groove are typical measurements, and the notch width index (NWI), the ratio of the NW to the bicondylar width, are most widely used in femoral notch size assessment. For more accurate assessment, the NW at different sites of the notch and corresponding NWI have been put forward. These measurements include the width at the notch inlet (NW_in), the width at the notch outlet (NW_ou), and the ACL attachment (NW_aa), etc. [12]. For many cases, the notch size can be efficiently evaluated by the NW and NWI [9, 13]. However, some other studies have presented the opposite results [9, 14]. One of the possible reasons for this discrepancy is that all of these 2D parameters can only denote the notch size of one location, which cannot adequately describe the 3-dimensional (3D) space.

The notch volume, which is a 3D notch parameter, describes the spatial dimensions of the femoral intercondylar notch and is an extension of the 2D parameters. Most studies, with the exception of one [13], have come to the conclusion that a smaller notch volume indicates a higher risk of ACL injury [8, 10, 11, 15]. However, due to the higher technical threshold and the more complicated measurement, the application of the notch volume measurement has been limited.

In this study, we researched the associations of the NW and NWI with the notch volume at four different locations (i.e., the popliteal groove, the notch inlet, the notch outlet, and the ACL attachment) of the femoral intercondylar notch in different groups (i.e., a male group, a female group, an ACL-injured group and an ACL-intact group). We attempted to confirm whether the common 2D notch parameters could be used to replace the 3D notch volumes in notch space assessment. Furthermore, the relationships of these parameters and non-contact ACL injuries were also assessed. We hypothesized that the typical 2D notch parameters could not represent the 3D notch volume, although most of these parameters could predict the risk of ACL injuries.

## Methods

### Sample size calculation, patient selection, and magnetic resonance images collected

The ethics of this retrospective case-control study were approved by our institutional research board. To determine the minimum number of participants required for our study, we performed a sample size calculation based on the results of van Eck et al. [9] (the Pearson correlation coefficient between the NW and notch volume was 0.551). To obtain a power of 0.99 (i.e., a β of 0.01) and an α of 0.01, we needed to include at least 53 participants in each group in the current study. We collected 240 participants in total (60 in each group) in this research, and the sample size reached the statistical requirement.

**Fig. 1:**
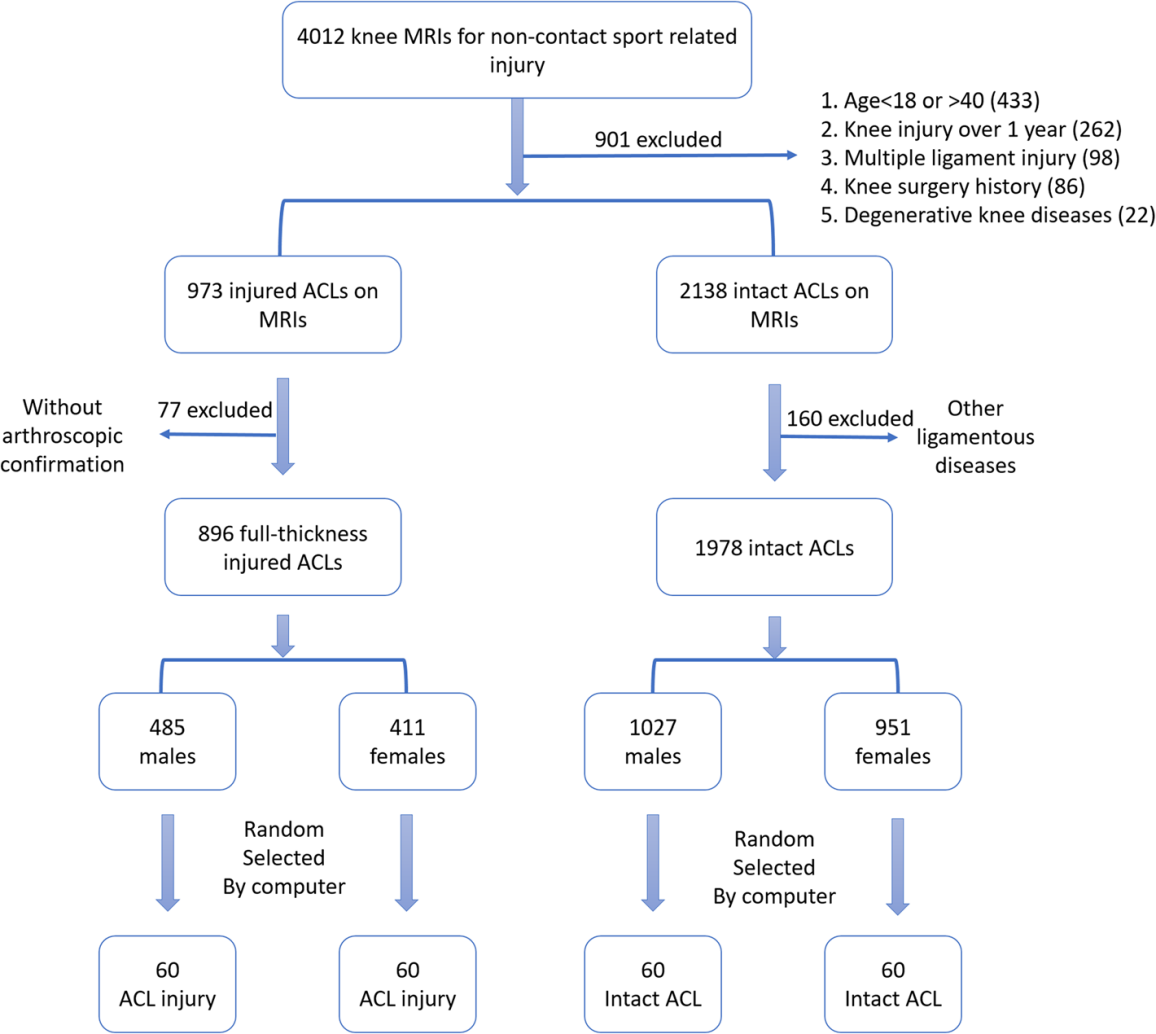
Flow chart of patient selection

We have found a total of 4012 newly diagnosed knee MRIs for non-contact sport related injury in our hospital from June 2015 to July 2016 (Fig. 1). We recruited only adults aged 18–40 years old for the femoral intercondylar notch measurements in this study. To avoid racial bias, all of the participants were Han Chinese. Juveniles were excluded due to their open epiphyseal plates and potential age-related changes in the femoral notches [10]. Due to the potential osteophytosis of the femoral notch that is caused by degenerative changes with age, middle-aged and elderly individuals were excluded. ACL-deficient individuals with knees that were hurt over one year before the MRI was collected were ruled out [11] because their knees might have degenerated much sooner than the normal, and osteophytosis of the notch might have affected the measurements of the NW, NWI, and notch volume. Additionally, patients with knee MRIs without clear femoral ACL attachments were also excluded.

We obtained 120 case-control pairs of individuals (60 males and 60 females) who were matched for gender and age. The demographic data (age, body height, and weight) were recorded. All of the ACL-injured patients (none of them with episodes of ACL injuries in bilateral knees) suffered first-time, sport-related, non-contact ACL injuries within one year. And the causes of their ACL injuries were listed in Table 1.To avoid false positive MRI results, all of the injuries were verified through ACL arthroscopy reconstruction surgeries. The patients with multiple ligament injuries or non-sport-related injuries were excluded to avoid evidence blurring. Those with contact ACL injuries were also ruled out because these injuries might have happened in high-energy impacts and been unrelated to any risk factors [16]. The ACL-intact group included individuals without any ligament injuries, such as synovitis, meniscus injuries, patellar tendinopathies or patellar dislocation. Those with knee injuries that were over one year old and those with previous operations were eliminated. Those with any diseases that might have altered the notch size were also excluded. These patients included those with knee degeneration, notch osteophytosis, and osteochondritis dissecans. All of the MRIs were obtained with the same MRI scanner machine (Siemens, Verio 3.0-T, slice thickness: 3.0 mm) in our hospital to avoid evident bias.

**Table 1:** The cause of non-contact ACL injuries in the research

Sport	Number of Cases	Number of Males	Number of Females
Basketball	28	18	10
Soccer	24	17	7
Skiing	13	5	8
Running	8	2	6
Tennis	12	5	7
Badminton	14	5	9
Others	21	8	13

### Notch width and notch width index measurements

The coronal plane of the knee joint was selected for measurement. The NWs at the four locations (i.e., the popliteal groove, the femoral notch inlet and outlet, and the ACL attachment) were measured, and the corresponding NWI was calculated.

**Fig. 2:**
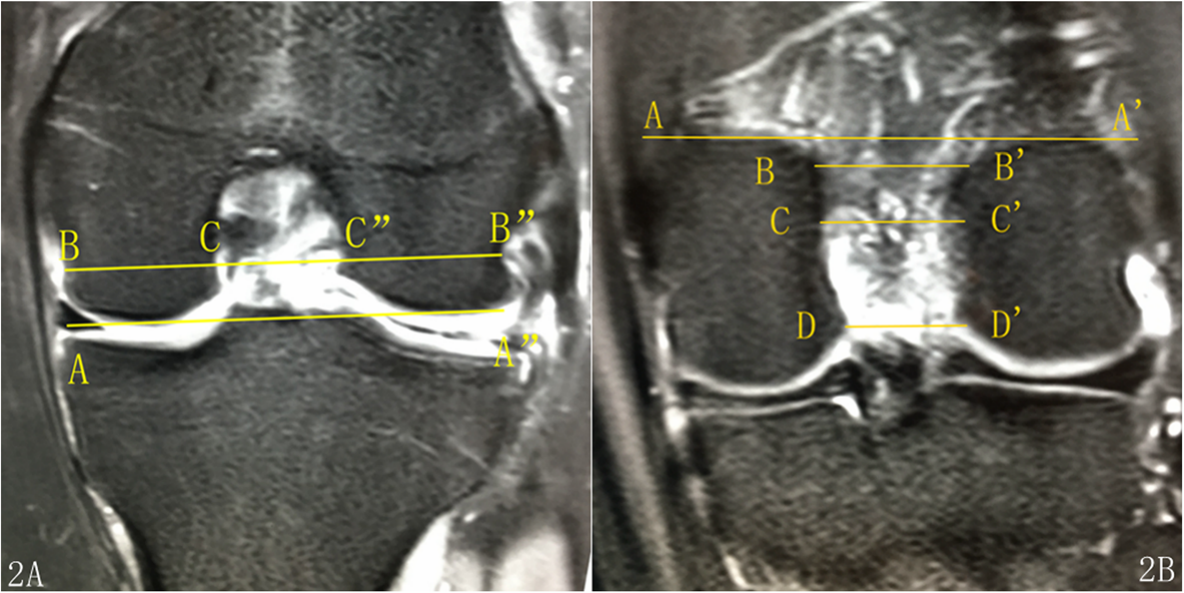
The coronal plane of the knee MRI shows the measurement of the NWs and the BW. 2A) AA” is the baseline, BB” is the BW at the level of the popliteal groove, CC” is the NW, and the NWI is equal to CC”/BB”*.* 2B) AA’ is the baseline, which is a proximal tangent of the lateral and medial femoral condyles. All the measurement of the notch width is in the same orientation as the baseline. BB’ is the NW at the notch inlet (NW_in), CC’ is the NW at the ACL attachment (NW_aa), DD’ is the NW at the notch outlet (NW_ou)

The measurements of the NW and NWI at the level of the popliteal groove were similar to those reported by van Eck et al. [9, 17]. The measurement slices were taken at the slice that exhibited a clear popliteal groove, and the reference line was marked as the distal tangent of the femoral condyles (Fig. 2a).

The measurements of the NW and NWI at the sites of the notch inlet, outlet, and ACL attachment were the same with those reported by Whitney et al. [12], which differed from the baseline selection of van Eck et al. The measurement plane was the coronal plane, which clearly displayed the notch inlet, outlet, and ACL attachment, and the reference line was marked as the proximal tangent of the femoral condyle (Fig. 2b).

All of the measurements were collected in the same manner by two experienced radiologists with the Tviews (WinningSoft, China) software, and the mean values were recorded.

### Notch volume measurement

**Fig. 3:**
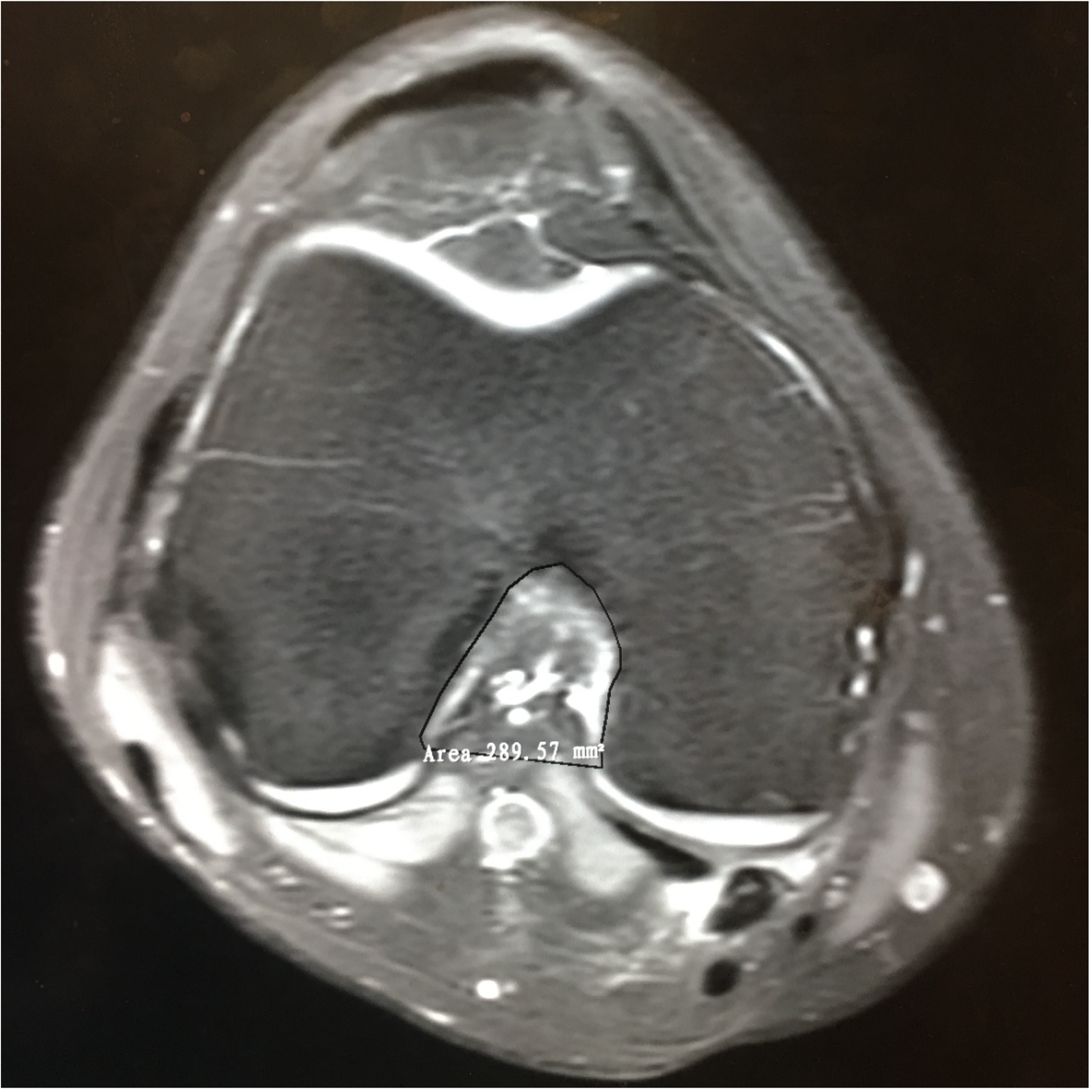
The axial slice of the knee MRI shows the measurement of femoral intercondylar notch volume. The black curve traces the boundaries of the femoral notch. And the number beside the tracing place is the area calculated by the software itself

The technique for the notch volume measurement was similar to that previously described by Charlton et al. [14], which was previously used in the ACL volume measurements in a porcine model [18]. First, we selected sequential 2D axial slices that displayed the boundaries of the femoral notch. Second, we manually traced the edges based on the anatomic landmarks, and the 2D notch area was calculated by automatically the software (Fig. 3). Third, we calculated the notch volume by summing all of the 2D areas multiplied by the slice thickness.

### Statistical analysis

For all of the demographic data and notch data, the means, standard deviations, and standard errors were calculated. The inter-observer homogeneity of the notch parameter measurements was examined with Cronbach’s α. Bivariate Pearson correlation coefficients were calculated to determine the correlations between the 2D parameters (NW and NWI) and the 3D notch volume. Multivariate conditional logistic regression analysis was conducted to display the independent effects of the NW, NWI, notch volume, age, body height, and weight on the ACL injuries. As the NW and NWI at the four sites might have been highly correlated, we examined only one of the eight relationships of the 2D notch parameters with the notch volume and demographic data in each of the multivariate logistic regressions, and we performed the multivariate logistic regressions eight times.

## Results

The number of individuals who were eligible according to the inclusion criteria was 240, and they were grouped according to gender and the presence of ACL injuries. The demographic data (age, height and weight), NW, NWI and notch volumes of the participants are displayed in Table 2, which includes the statistical results for the overall data and the subgroup data of the ACL-injured males, ACL-intact males, ACL-injured females and ACL-intact females.

**Table 2:** The demographic data and notch data of overall participants and subgroups

	Overall				Male Group		Female Group	
	Injury	Control	Males	Females	Injury	Control	Injury	Control
Number	120	120	120	120	60	60	60	60
Age (year)	29.78 ± 6.77	29.98 ± 5.94	28.94 ± 5.80	30.81 ± 6.76	28.15 ± 5.94	29.73 ± 5.58	31.4 ± 7.19	30.23 ± 6.30
Body Height (cm)	168.80 ± 8.68	168.32 ± 8.57	175.13 ± 5.32	162.00 ± 5.81	175.64 ± 4.79	174.62 ± 5.79	161.98 ± 5.84	162.02 ± 5.83
Body Weight (kg)	68.90 ± 14.77	68.26 ± 14.43	77.09 ± 12.41	60.07 ± 11.24	77.87 ± 12.14	76.31 ± 12.73	59.9 ± 11.35	60.21 ± 11.23
Time (month)^a^	3.37 ± 3.23	–	–	–	3.95 ± 3.68	–	2.79 ± 2.61	–
Notch Volume (cm^3^)	5.96 ± 1.38	6.84 ± 1.66	7.31 ± 1.58	5.49 ± 0.94	6.75 ± 1.30	7.88 ± 1.64	5.18 ± 0.95	5.81 ± 0.82
NW (mm)	20.17 ± 2.71	21.65 ± 2.70	22.19 ± 2.44	19.62 ± 2.55	21.49 ± 2.35	22.90 ± 2.34	18.84 ± 2.40	20.41 ± 2.47
NW_in(mm)	16.89 ± 2.57	19.62 ± 3.10	19.27 ± 3.18	17.23 ± 2.79	17.83 ± 2.51	20.72 ± 3.13	15.95 ± 2.28	18.52 ± 2.67
NW_aa(mm)	20.01 ± 2.46	21.12 ± 2.50	21.38 ± 2.57	19.75 ± 2.23	20.81 ± 2.42	21.94 ± 2.62	19.21 ± 2.25	20.29 ± 2.08
NW_ou(mm)	21.21 ± 5.50	22.91 ± 3.72	22.95 ± 3.73	20.76 ± 3.06	22.03 ± 2.99	23.86 ± 4.17	19.54 ± 2.70	21.97 ± 2.95
NWI	0.2921 ± 0.0309	0.3090 ± 0.0335	0.2963 ± 0.0299	0.3048 ± 0.0360	0.2901 ± 0.0294	0.3025 ± 0.0294	0.2940 ± 0.0326	0.3156 ± 0.0363
NWI_in	0.2517 ± 0.0747	0.2804 ± 0.0408	0.2581 ± 0.0391	0.2682 ± 0.0435	0.2418 ± 0.0316	0.2744 ± 0.0393	0.2501 ± 0.0375	0.2864 ± 0.0418
NWI_aa	0.2976 ± 0.0746	0.3022 ± 0.0340	0.2865 ± 0.0313	0.3070 ± 0.0318	0.2824 ± 0.0302	0.2906 ± 0.0321	0.3005 ± 0.0305	0.3138 ± 0.0321
NWI_ou	0.3288 ± 0.0291	0.3275 ± 0.0477	0.3072 ± 0.0443	0.3226 ± 0.0441	0.2988 ± 0.0371	0.3156 ± 0.0494	0.3058 ± 0.0385	0.3393 ± 0.0433

^a^The time of participation in physical and sports activities

The correlations between the 2D parameters (the NWs and NWIs at the four different sites) and the 3D femoral notch volume are displayed in Table 3, which includes the overall data analysis and the subgroup analyses. For all participants, the intercondylar notch volumes were moderately correlated with the NWs at the sites of the popliteal groove, notch inlet and ACL attachment, and minimally correlated with the NW at the notch outlet. In the subgroup analyses, the notch volume was irrelevant to the NWs at all of the four sites with the exception of the male ACL-intact group. The intercondylar notch volume was not related to the NWI at any of the four locations in the overall or subgroup analyses.

**Table 3:** The bivariate Pearson correlation coefficient between the notch volume and 2D notch parameters in overall and subgroup analysis

	Notch Volume				
	Overall	Male injured group	Male control group	Female injured group	Female control group
NW	0.453^b^	0.095	0.307^a^	0.113	0.177
NW_in	0.407^b^	0.193	0.256^a^	− 0.089	0.112
NW_aa	0.440^b^	0.125	0.404^b^	0.282^a^	0.262^a^
NW_ou	0.444^b^	0.241	0.387^b^	0.099	0.093
NWI	−0.013	−0.056	0.026	−0.035	0.029
NWI_in	0.036	0.088	0.045	−0.053	−0.031
NWI_aa	−0.072	−0.005	0.149	0.072	0.067
NWI_ou	0.064	0.143	0.232	0.035	−0.055

^a^Significant correlation at 0.05 level

^b^Significant correlation at 0.01 level

The associations of the 3D femoral notch volume and the 2D parameters (the NWs and NWIs at the four different sites) with the risk of ACL injury are displayed in Table 4. In both the male and female groups, the notch volume, NW, NW_in, and NW_aa were effective parameters for predicting the risk of a non-contact ACL injury (OR = 0.679, 0.844, 0.720 and 0.871, respectively). The NW_ou and NWI were effective parameters for ACL injury prediction only in the female group (OR = 0.765 and 0.836, respectively). The NWI_in, NWI_aa, and NWI_ou were ineffective for predicting ACL injury in the male and female groups.

**Table 4:** Multivariate Logistic Regression for Analysis of the Notch volume, 2D notch parameters, and demographic data^a^ in ACL injuries

Overall Data					Male Group			Female Group		
Variable	Unit	OR	95%CI	*P*-Value	OR	95%CI	P-Value	OR	95%CI	P-Value
Notch Volume^b^	1cm^3^	0.679	0.564–0.816	< 0.001	0.592	0.447–0.782	< 0.001	0.451	0.289–0.703	< 0.001
NW	1 mm	0.844	0.754–0.946	0.004	0.826	0.693–0.985	0.034	0.803	0.684–0.943	0.008
NW_in	1 mm	0.720	0.640–0.810	< 0.001	0.718	0.608–0.850	< 0.001	0.666	0.554–0.802	< 0.001
NW_aa	1 mm	0.871	0.770–0.985	0.028	0.888	0.752–1.048	0.156	0.845	0.699–1.019	0.096
NW_ou	1 mm	0.865	0.790–0.947	0.002	0.921	0.819–1.035	0.161	0.765	0.657–0.890	0.001
NWI	×100	0.862	0.788–0.943	0.001	0.884	0.769–1.016	0.082	0.836	0.744–0.939	0.030
NWI_in	×100	0.954	0.890–1.022	0.182	0.987	0.902–1.081	0.808	0.907	0.820–1.005	0.061
NWI_aa	×100	0.957	0.883–1.037	0.640	0.977	0.877–1.087	0.925	0.969	0.848–1.107	0.739
NWI_ou	×100	0.972	0.916–1.031	0.494	0.992	0.918–1.071	0.904	0.949	0.860–1.047	0.489

^a^The demographic data included the age, body height and weight; the results showed that the age and the body weight were not the risk factors of ACL injuries, the body height was the risk factor of ACL injuries for males, but not for females

^b^The notch volume here was the mean value of the eight times multivariate logistic regression analysis

OR: Multivariate odd ratio for ACL injuries in unit change

The mean inter-observer homogeneity (i.e., the mean Cronbach’s α value for all of the 2D parameters and the notch volumes) was 0.948.

## Discussion

One of the most important findings of our research is that the 3D femoral notch volume was almost irrelevant to the conventional 2D notch parameters, including the NW and NWI, at most of the four locations of the femoral notch in the subgroup analysis, which was in line with our hypothesis.

Although the notch volume and the NW were moderately correlated in the overall analysis, they were not correlated with each other in the subgroup analysis, with the exception of the male ACL-intact group. Therefore, the moderate correlation was probably caused by a confounding bias. Nevertheless, our results do not support the findings of van Eck et al. [9]. In their study, CT was applied for femoral notch volume measurement, and arthroscopy was used for the NW measurement, which resulted in a moderate correlation between the notch volume and the NW (including the notch inlet, the middle and the outlet). This was very important clinical research, although it was a study with a small sample study that included only 20 specimens who were not differentiated by gender, and older individuals with knee osteophytosis might not have been be ruled out. Additionally, the notch volumes were measured by CT, which neglected the notch cartilage, and the NWs were measured by arthroscopy, which was not sufficiently accurate due to location deviation. All of these factors blurred the evidence. The reason for the weak correlation between the NW and notch volume may be that the two parameters reflected different characteristics of the femoral notch. Although both parameters can reveal the notch size, the NW only represents the width of a specific location, whereas the notch volume demonstrates the overall size of the space.

Due to the convenience of measurement, 2D femoral notch parameters are the most widely used parameters. However, they only represent the size at one location of the notch and cannot adequately reflect the overall dimension [9], therefore, the measurement results may be biased. The 3D notch volume has advantages to describe the dimension of the femoral notch through 3D measurement. However, due to the higher technical threshold and the more tedious measurement, the application is limited.

We observed a severe lack of statistical correlation between the notch volume and the NWI, which is similar to the results reported by van Eck et al. [17]. One reason for the absence of a correlation between the notch volume and the NWI could be that the notch volume was an actual measurement of a 3D spatial dimension, whereas the NWI is the relative notch width as denoted by the ratio of the 2D notch width and the bicondylar width.

The notch volume and the NW at the popliteal groove, notch inlet, and ACL attachment were independent predictors of ACL injuries for both males and females based on the multivariate analyses of the data that were adjusted for age, body height and weight. Our results confirmed some of the results of previous research [8, 10–12, 19–21]. A decreased femoral notch size, including the notch volume and the notch widths at specific locations, increased the risk of suffering a non-contact ACL injury. The primary mechanisms include the ACL impingement theory and the small ACL hypothesis. The former means that the ACL might easily impinge on the inner wall of the lateral femoral condyle and lead to an ACL injury [2–4, 22], especially in the case of tibial plateau rotation shear stress from the front of the knee during flexion [4]. The later means that a small femoral notch size connotes a thin and vulnerable ACL, which could rupture in lower-load sports [14, 23].

Additionally, the NW at the femoral notch outlet, and the NWIs at the popliteal groove and femoral notch inlet were independent predictors of non-contact ACL injuries in the female group but not in the male group. Some other researchers have reported similar findings in that some parameters are useful for predicting ACL injuries in females but not in males [24]. The reasons remain unclear. Some scholars have indicated that the femoral notch size in females is smaller [10, 11, 13, 14], and the gender differences of notch anatomy [9] might be the explanation. Additionally, the ACL injury mechanisms seem to differ between males and females in previous studies [24]. Axial tibial compression combined with knee valgus loading and internal tibial rotation caused high tensile strain on the ACL [25], which might cause an ACL rupture at 15–27° of knee flexion according to research based on females [26].

Not all of the 2D femoral notch parameters were related to ACL injuries in our study. The NWIs at the locations of the femoral notch inlet, outlet, and ACL attachment were not effective parameters for predicting ACL injuries in the multivariate analysis. As the NWI is a ratio that reflects the relative stenosis of the femoral notch and has had numeric cut-off values from 0.19–0.27 in different studies [27–29], its accuracy might be insufficient. Moreover, we suspected that the relative stenosis of the femoral notch in some specific locations might not indicate ACL impingement. Furthermore, as the actual notch width and bicondylar width do not increase proportionately with body height, the validity of the NWI might be decreased [30]. Hence, the absolute parameters are occasionally more useful.

Notably, the absolute 2D notch parameters were not always effective for predicting ACL injuries [16, 31]. For example, the NW at the notch outlet was ineffective in the male group in our study. This result may be because some individuals have normal notch width values and narrow notch angles [16]. For example, the NW of a rounded base-stenotic notch may be normal, but the notch angle might not be. In other words, notch shapes are varied; a type A notch [28], which is associated with the morphology of high-risk of ACL injuries, could have the same notch width as other types of notches. Moreover, the 2D measurements are sensitive to rotation and angulation, and the accuracy is thus affected, which can be avoided in 3D analyses.

### Limitations

There are some potential limitations in our study. First, although the patient information was blinded, the researchers could specify the groups of the MRI because the status of the ACL was clearly displayed. This might have caused evidence blurring. To avoid this error, the measurements were performed twice by two experienced radiologists, and the high inter-observer homogeneity of the results demonstrated the reliability of our findings. Second, the individuals of the ACL-intact group were not the normal; they included patients suffering from meniscus injuries, synovitis, patellar tendinopathies and patellar dislocations, and these factors might have resulted in evidence bias. Moreover, no researchers have referred to this issue. To achieve more accurate measurements, we excluded the cases with bony defects or hyperostosis, such as osteochondritis dissecans, villonodular synovitis, and noticeable joint degenerative changes. Moreover, all of the MRIs were obtained from the same machine to prevent evidence bias. Third, our research only involved the Han Chinese, which account for more than 90% of the Chinese population. Since the anatomical differences of the human species exist objectively [32, 33], whether these results are established in all human beings needs further verification. Due to the lack of large-scale epidemiological data of ACL injury on the Chinese population, our research is a supplement to Chinese data.

## Conclusions

The current study demonstrated that there were very poor correlations between the characteristic 2D femoral notch parameters (including the NW and NWI) and the 3D notch volume. Therefore, the 2D notch parameters are not recommended as substitutes for the 3D measurements. The NWs at the popliteal groove, femoral notch inlet, outlet, and ACL attachment, and the NWIs at the popliteal groove and the notch volume were useful predictors of ACL injuries. For better femoral notch assessments, both 2D and 3D notch parameters are necessary.

## Abbreviation

2D: 2-dimensional
3D: 3-dimensional
ACL: Anterior cruciate ligament
MRI: Magnetic resonance imaging
NW: Notch width
NW_aa: The Notch width at the ACL attachment
NW_in: The Notch width at the notch inlet
NW_ou: The Notch width at the notch outlet
NWI: Notch width index
